# Validation of a modified Berger HIV stigma scale for use among patients with hepatitis C virus (HCV) infection

**DOI:** 10.1371/journal.pone.0228471

**Published:** 2020-02-05

**Authors:** M. Elle Saine, Tyler M. Moore, Julia E. Szymczak, Laura P. Bamford, Frances K. Barg, Nandita Mitra, Jason Schnittker, John H. Holmes, Vincent Lo Re

**Affiliations:** 1 Center for Clinical Epidemiology and Biostatistics, Center for Pharmacoepidemiology Research and Training, Department of Biostatistics, Epidemiology, and Informatics, Perelman School of Medicine, University of Pennsylvania, Philadelphia, PA, United States of America; 2 Leonard Davis Institute of Health Economics, University of Pennsylvania, Philadelphia, PA, United States of America; 3 Department of Psychiatry, Brain Behavior Laboratory, Perelman School of Medicine, University of Pennsylvania, Philadelphia, PA, United States of America; 4 Department of Medicine, Perelman School of Medicine, University of Pennsylvania, Philadelphia, PA, United States of America; 5 Philadelphia FIGHT Community Health Centers, Philadelphia, PA, United States of America; 6 Department of Family Medicine and Community Health, University of Pennsylvania Perelman School of Medicine, Philadelphia, PA, United States of America; 7 Department of Sociology, University of Pennsylvania, Philadelphia, PA, United States of America; Chinese Academy of Medical Sciences and Peking Union Medical College, CHINA

## Abstract

**Background:**

Stigma around hepatitis C virus (HCV) infection is an important and understudied barrier to HCV prevention, treatment, and elimination. To date, no validated instrument exists to measure patients’ experiences of HCV stigma. This study aimed to revise the Berger (2001) HIV stigma scale and evaluate its psychometric properties among patients with HCV infection.

**Methods:**

The Berger HIV stigma scale was revised to ask about HCV and administered to patients with HCV (n = 270) in Philadelphia, Pennsylvania. Scale reliability was evaluated as internal consistency by calculating Cronbach’s alpha. Exploratory factor analysis was performed to evaluate construct validity by comparing item clustering to the Berger HIV stigma scale subscales. Item response theory was employed to further evaluate individual items and to calibrate items for simulated computer adaptive testing sessions in order to identify potential shortened instruments.

**Results:**

The revised HCV Stigma Scale was found to have good reliability (α = 0.957). After excluding items for low loadings, the exploratory factor analysis indicated good construct validity with 85% of items loading on pre-defined factors. Analyses strongly suggested the predominance of an underlying unidimensional factor solution, which yielded a 33-item scale after items were removed for low loading and differential item functioning. Adaptive simulations indicated that the scale could be substantially shortened without detectable information loss.

**Conclusions:**

The 33-item HCV Stigma Scale showed sufficient reliability and construct validity. We also conducted computer adaptive testing simulations and identified shortened six- and three-item scale alternatives that performed comparably to the original 40-item scale.

## Introduction

Over 4.5 million people in the United States (US) have chronic hepatitis C virus (HCV) infection [1]. From 2010 to 2016, the incidence of HCV infection in the US increased 3.5-fold, driven largely by the opioid epidemic [2–4]. HCV-associated mortality now exceeds death from 60 other combined notifiable infectious diseases, including human immunodeficiency virus (HIV) infection [2]. Despite national action plans to eliminate HCV infection as a major public health threat by 2030 [5–7], fewer than half of HCV-infected persons in the US have been diagnosed or are aware of their infection [8], and treatment uptake remains <15% [9–13], undermining public health efforts.

Stigma is defined as a “deeply discrediting attribute” that differs from a *normal* attribute anticipated by society; functionally, stigma reduces “a whole and usual person to a tainted, discounted one” in the minds of dominant social groups [14]. Stigmatization is a social phenomenon that can be an immense burden for patients by impacting self-esteem, quality of life [15–17], and personal identity [18], ultimately impacting access to effective and equitable health care. Stigma can be psychologically taxing to patients, hindering them from seeking treatment, and inducing depression and/or self-isolation [19–21]. Clinical care that recognizes and addresses patient experiences of stigma can improve patient-provider communication, disease management, and health-related quality of life [6, 22–27].

HCV-related stigma has been identified as an important, but understudied, barrier to HCV treatment and elimination [8, 28–33]. Qualitative studies among patients with chronic HCV describe experiences of stigma in healthcare, such as poor patient-provider communication, insensitivity, and refusal of treatment [15, 17, 34]. However, to date, no validated scales exist to measure stigma among patients with HCV. Research that develops validated stigma measures and elucidates the nature of stigma is essential to patient-centered responses to public health. Improving understanding of HCV stigma can lead to interventions to increase rates of HCV diagnosis, linkage into care, and treatment. To address this knowledge gap, we revised the Berger HIV Stigma Scale (Berger-HSS) [35] to evaluate stigma associated with HCV infection. The Berger-HSS is a widely used measure of HIV stigma, which has been externally validated across HIV-infected patient populations within and outside of the US, [36–47] as well as adapted for use among non-HIV patient populations [48, 49]. We hypothesized that the revised HCV Stigma Scale would have good validity and reliability among patients with HCV infection due to similarities with HIV in modes of transmission and risk factors.

## Methods

### Instruments

The Berger-HSS [35] is a 40-item self-administered questionnaire using a four-point Likert scale with responses ranging from 1 (strongly disagree) to 4 (strongly agree). Two items (8 and 21) are reverse-scored. Scores are summed, with higher scores indicating greater experiences of stigma. Elements of the scale were adapted from the Rosenberg Self-Esteem Index [50], the Center for Epidemiologic Studies-Depression Scale [51], and the Multicenter AIDS Cohort Study/Coping and Change Study [52] to asses four factors of stigma: 1) personalized stigma; 2) fear of disclosure; 3) negative self-image; and, 4) concern about public attitudes. The Berger-HSS was developed and validated among 318 patients with HIV infection in the Midwestern US. The scale demonstrated excellent internal consistency (Cronbach α, 0.90–0.93) and test-retest correlation (0.89–0.92), and is widely considered the benchmark instrument for assessing HIV stigma among people living with HIV/AIDS [53], primarily due to its stable external validity across diverse populations of patients [37–39, 54, 55]. Modified/shortened versions have also been validated across HIV-infected populations [42, 44–47, 56, 57]. For this study, we revised the Berger-HSS to ask about HCV-related stigma (HCV-SS), by replacing “HIV” with “Hepatitis C” in all instructions and questions. No other revisions to the Berger-HSS were made.

From July 2018 to May 2019, HCV-SS instruments were administered on laptop computers equipped with headphones, using audio computer-assisted self-interview software (ACASI). ACASI has been used extensively in healthcare studies to improve data quality and minimize social desirability bias around sensitive topics and risk behaviors, including stigma, knowledge, and disease experience [37, 43, 58–73]. Each response set includes the options “I don’t know the answer” and “I don’t want to answer.” A free-text response box following the HCV-SS allowed participants to share any additional information that they felt was important or not collected by other survey questions.

### Study design and setting

We conducted a cross-sectional study among patients presenting for care at Philadelphia outpatient clinics specializing in the treatment of HCV infection. From 2013–2016, the rate of HCV infection in Philadelphia was among the highest in the US, with over 410 cases per 100,000 people [2]. Participants were recruited from five clinics across two Philadelphia health systems: 1) University of Pennsylvania Health System (UPHS); and, 2) Philadelphia Field Initiation Group for HIV Trials (FIGHT) Community Health Centers.

UPHS is a nationally-ranked academic health system providing outpatient specialty HIV and viral hepatitis care. Patients were recruited from two UPHS clinics: 1) Center for Viral Hepatitis at Penn Presbyterian Medical Center (PPMC); and, 2) Hospital of the University of Pennsylvania MacGregor Infectious Diseases Clinic (HUP). The demographic characteristics of patients within the UPHS service area are largely representative of the Philadelphia patient population [74].

Philadelphia FIGHT is a Federally-Qualified Health Center providing comprehensive health services to individuals with low income, people living with HIV/AIDS, and those at high risk of contracting HIV/AIDS.[75] Patients were recruited from three FIGHT clinics: 1) Jonathan Lax Treatment Center (LAX); 2) John Bell Health Center (JBHC); and, 3) Clinica Bienestar. LAX and JBHC provide primary medical care to adult patients ≥18 years of age. Clinica Bienestar is a comprehensive HIV primary care clinic in partnership with and housed within Prevention Point Philadelphia, Philadelphia’s only syringe-service program. Clinica Bienestar providers specialize in the care of Puerto Rican patients and patients with active or previous injection drug use. The clinic has been recognized as an Innovative Practice by the Health Resources and Service Administration [76]. The demographics of the Philadelphia FIGHT patient population are largely representative of the larger population of chronic HCV-infected patients in Philadelphia [3, 76].

This study was approved by the Institutional Review Boards of both the University of Pennsylvania and Philadelphia FIGHT.

### Participants and recruitment

Patients were eligible for study inclusion if they were identified by their provider to be: 1) ≥18 years of age at enrollment; 2) positive for HCV antibody; and, 3) English-speaking. The HCV antibody test criterion was chosen to include patients who had ever been diagnosed with HCV infection, including patients who have chronic HCV, spontaneously cleared the virus, or cured with antiviral treatment. All eligible patients were invited to participate until the desired sample size was reached.

### Data collection

During survey administration, we also collected self-reported sex, gender identity, race, ethnicity, age, HIV coinfection status, and stage of HCV-management.

### Statistical analysis

Baseline demographic and clinical characteristics were collected as categorical variables and presented descriptively as counts and proportions. Descriptive statistics were calculated overall and by HIV coinfection status. Covariate differences by HIV coinfection status were assessed using chi-square and Fisher’s exact tests, as appropriate.

Because the item responses are on an ordinal scale, we calculated the polychoric correlation matrix, setting negative eigenvalues to zero to obtain the least-squares positive semidefinite approximation of the matrix. We inspected the correlation matrix to confirm that variables within a hypothesized factor were moderately correlated (≥0.3), and a Kaiser-Meyer-Olkin (KMO) measure of sampling adequacy was performed to ensure that the data were suitable for factor analysis. Consistent with Berger et al. [35], we performed exploratory factor analysis (EFA) to evaluate the latent structure of the items, allowing items to freely load on all factors [35]. We employed the iterated principal factor extraction method and inspected the pattern matrix for Heywood cases [77, 78].

**Fig 1:**
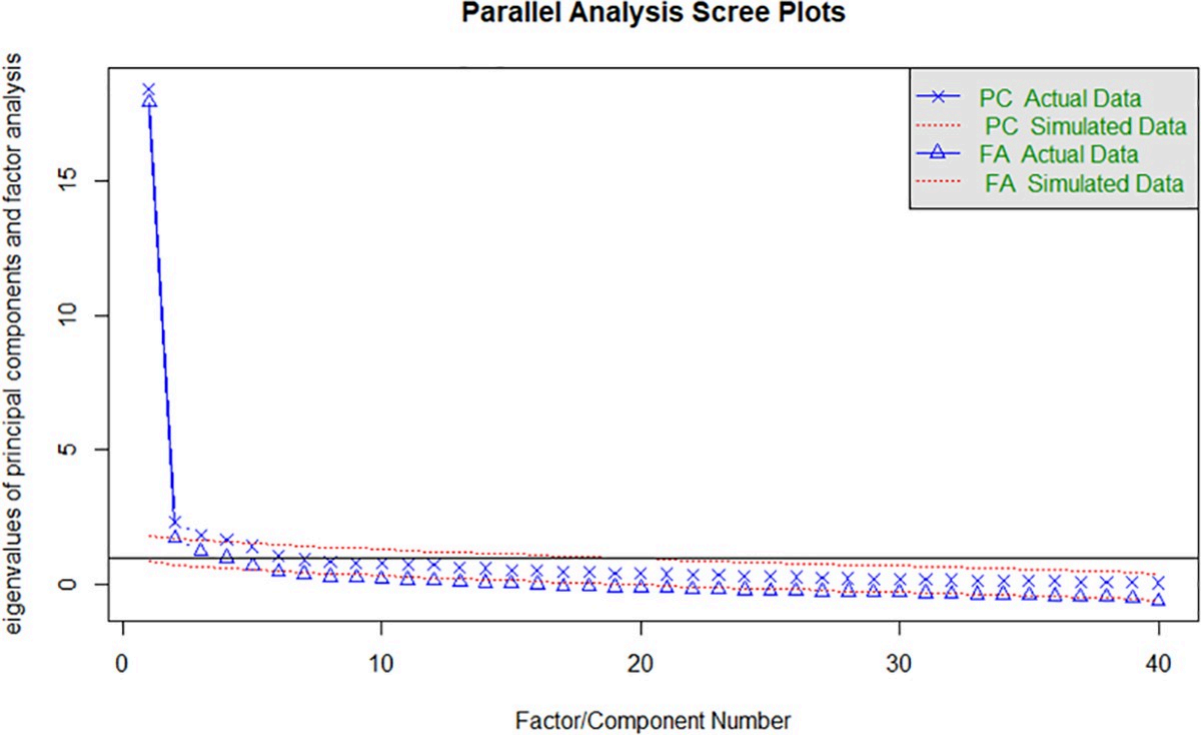
Parallel analysis scree plot of eigenvalues. Scree plot of actual and simulated data for factor analysis (FA) and principal component (PC) analyses.

We evaluated the number of factors using multiple approaches: 1) visual examination of the scree plot; 2) Kaiser Criterion of eigenvalues ≥ 1.0 [78]; 3) parallel analysis comparing the progressive eigenvalues of the dataset to eigenvalues from randomly generated data of the same dimensions in order to account for variance due to chance [77, 78]; 4) the Very Simple Structure Criterion, which compares the correlation matrix to a simplified model composed of the largest loadings for each variable [79]. While the Kaiser Criterion and parallel analysis extracted a four-factor solution, the Very Simple Structure Criterion suggested a single factor solution. Moreover, as shown in the scree plot (**Fig 1**), the first factor explains a substantial proportion of the variance, with a 1^st^/2^nd^ eigenvalue ratio of 9.64, indicating that a single factor (unidimensional) solution may be appropriate. We therefore evaluated a four-factor solution and a one-factor solution.

Items were assigned to a factor if they loaded at ≥0.4 [78]. We assessed reliability by calculating the internal consistency, estimated by Cronbach α, for the scale and each subscale, with a sufficient Cronbach α ≥0.7 [80, 81]. We investigated the absence of floor and ceiling effects, defined as 15% or more of the respondents with the lowest or highest possible score, as an estimate of content validity.

The four-factor solution allows replication of subscale analyses by Berger to evaluate item clustering as a measure of construct validity [35]. Construct validity, defined as the extent to which items in a scale are consistent with theoretical hypotheses regarding the phenomenon being measured [80], was determined by ≥75% of items loading on the same factor as in the Berger-HSS [80]. Items in our analyses with two or more loadings at ≥0.4 were considered cross-loading items and were assigned to the highest loading factor. For cross-loading items in the Berger-HSS, if the HCV-SS item loaded on at least one of the predefined Berger factors, it was considered to support the construct validity of the scale. The explained common variance of the four-factor solution was calculated using minimum rank factor analysis.

In order to evaluate individual scale items, we chose to further analyze the single factor solution using item response theory (IRT). IRT assumes that the unidimensional latent trait under study exists on a continuum and allows investigation of how each item performs among participants at varying levels of the underlying trait [2]. A key benefit of IRT is that it allows evaluation of the amount of information provided by each individual scale items, thereby assessing the quality of each item [82]. Moreover, IRT allows evaluation of shortened scales using computer adaptive testing (CAT).

Items were calibrated using the IRT-graded response model [83], an extension of the traditional 2-parameter (2PL) model, to handle ordered polytomous categories, such as the Likert scale. Given an individual’s trait level (θ), the probability of a participant selecting item response *k* or higher for item *i* is dependent on two parameters: 1) discrimination (*𝑎*_*i*_), can be thought of as a measure of an item’s precision in placing an individual on the trait spectrum; and, 2) difficulty (*𝑏*_*i*_), is the probability that an individual at a specific point on the trait spectrum would endorse an item. The graded response model is defined by the following equation:

$$P_{ik}^{*}(\theta )=\frac{exp(a_{i}[\theta -b_{ik}])}{1+exp(a_{i}[\theta -b_{ik}])}$$

Where for item *I*, *𝑎*_*i*_ is the discrimination parameter and *b*_*ik*_ is the difficulty parameter for each response category *k*. The amount of information that an item provides increases as the item discrimination increases, and is maximized when the difficulty equals an individual’s trait level (when *θ* = *b* in the above equation) [2, 82–85].

Because the Berger-HSS was developed among patients with HIV infection, we conducted differential item functioning analyses using the *lordif* package [79] in R (R Core Team, 2016) in order to evaluate whether the probability of endorsement for any HCV-SS items differed between participants who were monoinfected with HCV compared to those coinfected with HIV/HCV. Differential item functioning analyses were conducted via ordinal logistic regression, with the graded response model used for trait estimation and the theta estimated as the conditioning variable. Items were flagged when differences in difficulty and discrimination parameters were detected between subgroups of the study population. Differential item functioning analyses allow the elimination of items that may provide misleading results, analogous to effect modification or confounding [86].

Using both a ratio of five participants for each measured item and the assumption of moderate conditions (communalities of 0.40–0.70 and ≥3 measured variables loading on each factor), a sample size of ≥200 participants was considered sufficient for exploratory factor analyses [78, 80]. For IRT analyses, Chang et al demonstrated that a sample size ≥250 participants can produce reasonable estimates for a 4 point Likert scale like ours, although ≥500 participants are required for accurate parameter estimates when using an IRT model with a five-point Likert scale [87–90]. There was less than 5% missingness on items; missing data were imputed using multiple imputation by chained equations. Data were analyzed using Stata 15.0 (Stata Corporation, College Station, TX) and the *mirt* [91], *psych* [79] and *lordif* [79] packages in R (R Core Team, 2016).

### Shortening of HCV-SS

To optimize efficiency of the HCV-SS, we simulated computerized adaptive testing (CAT) to determine, 1) if a subset of items could be used in a shortened form, and if so, 2) which items an optimal subset would comprise. Briefly, CAT determines which item in the question bank will provide the most information about a participant’s trait level by roughly estimating the trait level based on the participant’s response to the administered item and then choosing a subsequent item that will provide the most additional information. The algorithm updates information on the participant as she/he responds to each question and stops when the participant’s standard error of measurement (SEM) reaches a predetermined lower bound. CAT minimizes test burden by omitting items that provide little information about a participant and retaining items that are the most informative [19, 82, 92].

Estimated item parameters from the one-factor graded response model were imputed into Firestar [93], a CAT simulation program. All simulations were performed using real data simulation. The first administered item was selected based on maximum information at the mean. The default settings were kept as maximum posterior weighted information for the item selection method and the interim theta estimator was expected *a posteriori* [82, 94, 95]. Two CAT simulations were run, first with the stopping rule set at a SEM≤0.3, and second with the stopping rule set at SEM≤0.4. SEM of 0.3 and 0.4 are equivalent to an alpha 0.91 and 0.84 respectively, using the classical test theory conversion of α = 1 –SEM^2^ [82]. The output of the simulation program indicates the average number of items administered across simulations and the distribution of item usage in order to identify shortened scales composed of the most useful items.

## Results

### Patient characteristics

We approached 288 patients with a history of a positive HCV antibody test, of whom 270 (96.43%) agreed to participate in the survey. 265 participants completed the survey, all of whom had sufficient data for analyses (<5% missing data). Participants were predominantly male (68.68%), 55 years of age or older (35.47%), and white (46.79%) or black/African American (39.25%). Most participants had been diagnosed with HCV infection more than one year prior to the survey (81.13%) and were either currently being treated (29.06%) or cured (41.51%) of chronic HCV infection. The total instrument took an average of 16 minutes to complete, with the stigma scale alone taking 10 minutes.

Slightly more than half of participants were coinfected with HIV (147, 55.47%). Compared to HCV-monoinfected participants, HCV/HIV-coinfected participants were older (P = 0.001), more frequently identified as black/African American (P<0.001), and had a higher rate of HCV treatment and cure (P<0.001; **Table 1**).

**Table 1:** Baseline characteristics of participants, overall and by HIV infection status.

Characteristics*	Overall (n = 265)	HCV-Monoinfected (n = 118)	HCV/HIV-Coinfected (n = 147)	P-Value
**Age, years (n, %)**				
18–24	3 (1.13%)	1 (0.85%)	2 (1.36%)	**0.001**
25–34	34 (12.83%)	22 (18.64%)	12 (8.16%)	
35–44	63 (23.77%)	36 (30.51%)	27 (18.37%)	
45–54	71 (26.79%)	30 (25.42%)	41 (27.89%)	
55+	94 (35.47%)	29 (24.58%)	65 (44.22%)	
**Sex/gender identity (n, %)**				
Male	182 (68.68%)	87 (73.73%)	95 (64.63%)	0.21
Female	75 (28.30%)	30 (25.42%)	45 (30.61%)	
Male identifying as female/Transgender/Transsexual	4 (1.51%)	0 (0.00%)	4 (2.72%)	
Female identifying as male/Transgender/Transsexual	2 (0.75%)	1 (0.85%)	1 (0.68%)	
Queer / gender non-conforming	1 (0.38%)	0 (0.00%)	1 (0.68%)	
Other / prefer not to answer	1 (0.38%)	0 (0.00%)	1 (0.68%)	
**Race (n, %)**				
White	124 (46.79%)	73 (61.86%)	51 (34.69%)	**<0.001**
Black / African American	104 (39.25%)	32 (27.12%)	72 (48.98%)	
Hawaiian / Pacific Islander	14 (5.28%)	4 (3.39%)	10 (6.80%)	
American Indian / Alaskan Native	10 (3.77%)	4 (3.39%)	6 (4.08%)	
Multiracial	7 (2.64%)	3 (2.54%)	4 (2.72%)	
Other / Prefer not to answer	6 (2.26%)	2 (1.69%)	4 (2.72%)	
**Ethnicity (n, %)**				
Not Hispanic / Latino	205 (77.36%)	96 (81.36%)	109 (74.15%)	0.21
Hispanic / Latino	57 (21.51%)	20 (16.95%)	37 (25.17%)	
Other / Prefer not to answer	3 (1.13%)	2 (1.69%)	1 (0.68%)	
**History of injection drug use (n, %)**	196 (73.96%)	91 (77.12%)	105 (71.43%)	0.29
**Time since HCV diagnosis (n, %)**				
Diagnosed within previous year	37 (14.68%)	22 (19.64%)	15 (10.71%)	0.145
1–5 years	75 (29.76%)	37 (33.04%)	38 (27.14%)	
6–10 years	45 (17.86%)	18 (16.07%)	27 (19.29%)	
11–15 years	34 (13.49%)	15 (13.39%)	19 (13.57%)	
16–20 years	34 (13.49%)	11 (9.82%)	23 (16.43%)	
21–25 years	16 (6.35%)	7 (6.25%)	9 (6.43%)	
>25 years	11 (4.37%)	2 (1.79%)	9 (6.43%)	
**Stage of HCV Management (n, %)**				
Spontaneously cleared	13 (4.91%)	6 (5.08%)	7 (4.76%)	**<0.001**
Diagnosed, Untreated	48 (18.11%)	27 (22.88%)	21 (14.29%)	
Previously treated, Not cured	17 (6.42%)	8 (6.78%)	9 (6.12%)	
Currently being treated	77 (29.06%)	52 (44.07%)	25 (17.01%)	
Treated, Cured	110 (41.51%)	25 (21.19%)	85 (57.82%)	

Abbreviations: HIV = Human Immunodeficiency Virus; HCV = Hepatitis C Virus; Trans = Transgender

* Characteristics are presented as percentages.

### Exploratory factor analyses

The data were found to be suitable for EFA with a KMO of 0.913. Floor and ceiling effects were absent.

For the four-factor solution, six items (5, 8, 11, 19, 21, 22) had loadings below the 0.40 cutoff used here and were removed from subsequent EFA analyses. Item clustering for the 34 (85%) items with loadings ≥0.40 matched the Berger-HSS four-factor EFA [35], providing evidence for the construct validity of the sub-scales among patients with HCV. Additionally, two items (26 and 37) cross-loaded on factors 1 and 2; both items had the highest loading on factor 2 and were therefore assigned to this factor (**Table 2**).

**Table 2:** Exploratory factor analysis of four- and one-factor solutions and graded response model parameter estimates of one-factor solution. Factor loadings presented include four-factor and one-factor solutions. Bolded loadings indicate agreement of factor loadings in four-factor solution with Berger Subscales*.

Item	Four-Factor Solution				One-Factor Solution					Berger Subscale
	Factor Loading				Factor Loading	Difficulties			Discrimination^†^	
	F 1	F 2	F 3	F 4		b_1_	b_2_	b_3_		
1	-0.006	**0.524**	0.036	-0.315	0.105	-10.527	-3.894	8.189	0.179	2
2	-0.054	0.096	**0.718**	-0.087	0.479	-0.886	1.019	3.361	0.928	3
3	0.089	0.197	**0.640**	0.008	0.705	-0.841	0.480	1.747	1.692	3
4	-0.118	**0.651**	0.223	0.104	0.557	-1.588	-0.184	1.845	1.141	2, 4
5	0.091	0.173	0.163	0.314	0.575	-0.906	1.077	2.905	1.197	4
6	0.179	**0.413**	0.268	0.012	0.675	-1.101	0.392	1.812	1.558	2, 3
7	0.032	-0.017	**0.639**	0.121	0.590	-0.109	1.342	2.911	1.243	3
8^‡^	0.066	-0.059	0.208	-0.066	0.175	-5.189	-0.162	5.785	0.302	3
9	0.148	0.029	0.019	**0.620**	0.658	-1.299	0.469	2.293	1.488	4
10	-0.095	0.140	0.115	**0.695**	0.603	-1.746	-0.407	1.749	1.287	4
11	0.118	0.389	0.195	0.175	0.667	-1.123	0.320	1.771	1.525	2, 3, 4
12	0.112	-0.042	**0.594**	0.267	0.744	-0.595	0.649	2.069	1.896	3
13	0.166	-0.081	**0.607**	0.221	0.742	-0.508	0.934	2.135	1.883	1, 3, 4
14	0.030	-0.019	0.335	**0.594**	0.717	-1.228	0.352	1.806	1.753	4
15	0.289	-0.095	**0.517**	-0.079	0.581	-0.275	1.652	3.611	1.214	3
16	0.361	0.031	0.024	**0.563**	0.800	-1.351	0.101	1.569	2.268	1, 4
17	0.013	**0.651**	-0.052	0.278	0.578	-2.358	-1.148	0.842	1.207	2
18	**0.488**	0.037	0.128	0.137	0.735	-0.984	0.649	2.049	1.844	1
19	0.345	0.249	0.155	0.255	0.818	-1.184	-0.047	1.225	2.421	2, 4
20	0.257	0.222	0.032	**0.454**	0.744	-1.423	0.039	1.805	1.897	4
21^‡^	-0.202	0.399	0.304	-0.136	0.208	-5.896	-1.231	4.751	0.362	2
22	0.233	0.378	0.078	0.343	0.774	-1.586	-0.466	1.214	2.083	2, 4
23	0.221	0.103	**0.496**	-0.009	0.689	-0.873	0.511	2.054	1.619	3
24	**0.534**	0.153	0.042	0.232	0.842	-0.932	0.203	1.365	2.653	1
25	0.338	**0.445**	0.088	0.121	0.784	-1.358	-0.148	1.139	2.153	2
26	**0.428**	0.434^§^	0.011	0.051	0.764	-1.404	0.043	1.414	2.014	1
27	**0.429**	0.335	0.082	0.098	0.779	-1.247	0.246	1.355	2.113	1, 3, 4
28	**0.749**	0.009	0.004	0.111	0.853	-0.965	0.260	1.411	2.782	1, 4
29	**0.806**	-0.011	0.139	-0.147	0.827	-0.568	0.829	1.849	2.504	1
30	**0.543**	-0.069	0.315	-0.006	0.764	-0.763	0.514	1.794	2.015	1, 4
31	**0.865**	0.016	-0.004	-0.086	0.825	-0.710	0.499	1.824	2.483	1
32	**0.826**	-0.066	0.085	0.022	0.875	-0.718	0.324	1.336	3.081	1, 4
33	**0.861**	0.050	0.005	0.025	0.908	-0.815	0.358	1.578	3.685	1, 4
34	**0.525**	0.019	0.052	0.162	0.704	-1.402	-0.273	1.494	1.688	1, 4
35	**0.779**	-0.009	-0.031	0.135	0.868	-0.759	0.316	1.539	2.975	1
36	**0.859**	-0.089	0.013	0.039	0.858	-0.677	0.552	1.614	2.849	1
37	0.450	**0.551**^§^	-0.055	-0.003	0.770	-1.098	0.105	1.356	2.057	2
38	**0.758**	0.082	0.053	-0.049	0.827	-0.721	0.613	1.859	2.506	1, 3, 4
39	**0.813**	0.042	-0.013	0.028	0.865	-0.723	0.415	1.577	2.928	1, 3, 4
40	**0.644**	0.091	0.049	0.069	0.802	-1.161	0.274	1.851	2.284	1, 4

*Shaded boxes indicate factor loading ≥0.4; bolded text indicates that item clustering corresponds with Berger HIV Stigma Scale

^†^IRT parameters are in the logistic metric (D = 1.0)

^‡^Reverse-scored item

^§^Assigned factor in exploratory factor analysis for cross-loading items

The four-factor 34-item scale was determined to be reliable with a Cronbach α of 0.957. All four factors also had sufficient reliability with Cronbach α results ranging from 0.819–0.946. The inter-factor correlations (range, 0.371 to 0.599) indicate that, in addition to inter-item covariance being explained by individual factors, there exists a strong underlying factor (stigma) that explains a large portion of the covariance among all items (**Table 3**). The explained common variance of the four-factor solution was 59.0%.

**Table 3:** Inter-factor correlations and reliability of four-factor solution*.

Scale	Inter-Factor Correlations				Number of Items	Cronbach α
	1	2	3	4		
Four-Factor Scale	-				34	0.957
1. Personalized Stigma Subscale	-				15	0.946
2. Disclosure Subscale	0.441	-			7	0.819
3. Negative Self-Image Subscale	0.599	0.364	-		7	0.846
4. Public Attitudes Subscale	0.529	0.371	0.433	-	5	0.843

*Subscales of four-factor solution, labeled according to the Berger HIV Stigma Scale Subscales

Three items (1, 8, 21) had loadings <0.4 on the one-factor solution. Factor loadings are presented in **Table 2**. The one-factor model was determined to be reliable with a Cronbach alpha of 0.963 and explained 51.8% of the variance.

### Item response theory analyses

The one-factor solution was further evaluated using item response theory. Mean discrimination for the total scale was 1.894 (range, 0.179 to 3.685). Difficulty parameters varied across the polytomous response categories, with the lowest threshold (b_1_) indicating the “easiest” (mean -1.489; range -10.527 to -0.109), difficulty progressing as level of stigma endorsement (b_2_) increased (mean 0.189; range -3.894 to -1.652), and the upper thresholds (b_3_) all positive (mean 2.146; range 0.842 to 5.785; **Table 2**). Five items (2, 8, 12, 22, 36) were flagged for differential item functioning between patients monoinfected with HCV vs coinfected with HCV/HIV. Items with low factor loadings and those flagged for differential item functioning were removed, resulting in a 33-item scale (**S1 Appendix**).

### Shortened HCV-SS scales

**Fig 2:**
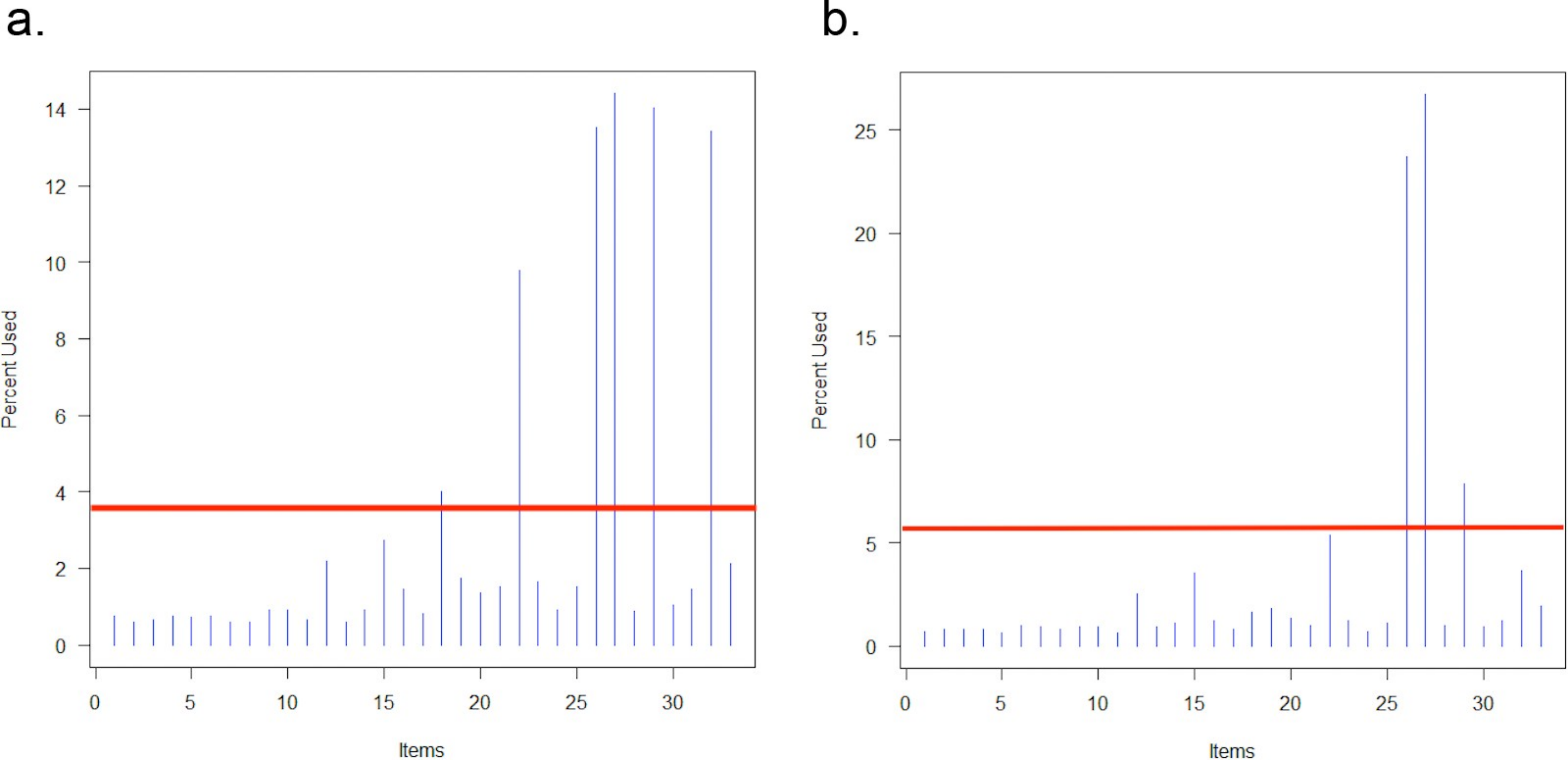
Histogram of item usage for adaptive simulation with standard error of measurement ≤0.3 (a) and ≤0.4 (b).

Simulated CAT using item parameters estimated in the 33-item sample yielded a six-item scale (items 24, 28, 32, 33, 35, 39) when using a stopping criterion of SEM≤0.3 and a three-item scale (items 32, 33, 35) when using a stopping criterion of SEM≤0.4. **Fig 2** shows the percent of total items used on the y-axis, which is calculated by taking the total number of times each item was administered, divided by the total number of items administered across all examinees; this adds up to 100% for all items. Specifically, item 33 provides enough information that it was always administered, regardless of the examinee’s level of stigma; similarly, items 28, 32, 35, and 39 were administered in most simulations.

**Fig 3:**
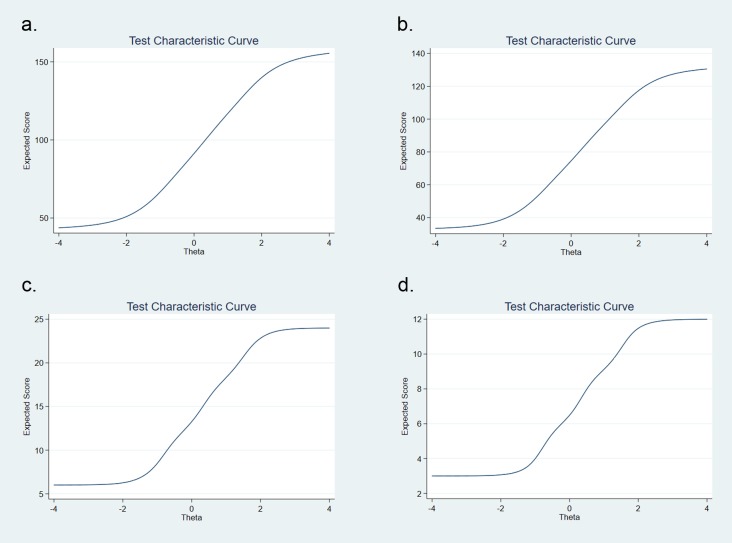
Test characteristic curves for total scale and short forms. Comparison of test characteristic curves (TCC) for full 40-item scale (a) with full 33-item HCV Stigma Scale (b), 6-item (c), and 3-item (f) scales.

Both the six-item and three-item shortened scales demonstrated good internal consistency reliability (0.909 and 0.861, respectively). After correcting for item redundancy using Levy’s formula,[96] scores for the six-item and three-item shortened scales demonstrated good correlation (>80%) with scores from the 40-item scale (**Table 4**). Furthermore, the test characteristic curves for the short scales all had nearly identical shapes to the 40-item Berger HSS and 33-item full HCV-SS (**Fig 3**).

**Table 4:** Item selection and correlation of 33-item scale and shortened 6-item and 3-item scales with Berger 40-Item Scale.

		Shortened Scales		
		33 Item^†^	6 Item^‡^	3 Item^§^
**Cronbach’s α**		0.959	0.909	0.861
**Correlation with 40 Item Berger Scale**		0.959	0.877	0.838
**Item Usage**				
** Item**	Question*			
1	In many areas of my life, no one knows that I have HCV			
2	I feel guilty because I have HCV			
3	People's attitudes about HCV make me feel worse about myself	S		
4	Telling someone I have HCV is risky	S		
5	People with HCV lose their jobs when their employers find out	S		
6	I work hard to keep my HCV a secret	S		
7	I feel I am not as good a person as others because I have HCV	S		
8	I never feel ashamed of having HCV^R^			
9	People with HCV are treated like outcasts	S		
10	Most people believe that a person who has HCV is dirty	S		
11	It is easier to avoid new friendships than worry about telling someone that I have HCV	S		
12	Having HCV makes me feel unclean			
13	Since learning I have HCV, I feel set apart and isolated from the rest of the world	S		
14	Most people think that a person with HCV is disgusting	S		
15	Having HCV makes me feel that I'm a bad person	S		
16	Most people with HCV are rejected when others find out	S		
17	I am very careful who I tell that I have HCV	S		
18	Some people who know I have HCV have grown more distant	S		
19	Since learning I have HCV, I worry about people discriminating against me	S		
20	Most people are uncomfortable around someone with HCV	S		
21	I never feel the need to hide the fact that I have HCV^R^	S		
22	I worry that people may judge me when they learn I have HCV			
23	Having HCV in my body is disgusting to me	S		
24	I have been hurt by how people reacted to learning I have HCV	S	S	
25	I worry that people who know I have HCV will tell others	S		
26	I regret having told some people that I have HCV	S		
27	As a rule, telling others that I have HCV has been a mistake	S		
28	Some people avoid touching me once they know I have HCV	S	S	
29	People I care about stopped calling after learning I have HCV	S		
30	People have told me that HCV is what I deserve for how I lived my life	S		
31	Some people close to me are afraid others will reject them if it becomes known that I have HCV	S		
32	People don't want me around their children once they know I have HCV	S	S	S
33	People have physically backed away from me when they learn I have HCV	S	S	S
34	Some people act as though it's my fault I have HCV	S		
35	I have stopped socializing with some people because of their reactions to my having HCV	S	S	S
36	I have lost friends by telling them I have HCV			
37	I have told people close to me to keep the fact that I have HCV a secret	S		
38	People who know I have HCV tend to ignore my good points	S		
39	People seem afraid of me once they learn I have HCV	S	S	
40	When people learn you have HCV, they look for flaws in your character	S		

S = Item selected in scale

*HCV was written as “hepatitis C virus” in the administered scale

^†^Removed items with insufficient loading and/or differential item functioning (DIF)

^‡^Adaptive test simulation with stopping criteria of standard error of measurement (SEM) < 0.30

^§^Adaptive test simulation with stopping criteria of standard error of measurement (SEM) < 0.40

^R^Reverse scored item

## Discussion

Stigma, along with other forms of social marginalization, can influence a patient’s decision to undergo testing, his/her adherence to treatment, and retention in health care globally [97]. Understanding how perceptions of stigma influence decision-making among HCV-infected patients is essential to understanding barriers to healthcare among this population and to planning interventions to address the public health threat of this disease. To our knowledge, this is the first study to validate a scale to measure perceptions of stigma among patients with HCV infection. Development of a validated questionnaire increases precision in understanding patients’ perspectives on HCV-related stigma and its impact on HCV diagnosis, retention in care, and antiviral treatment [18, 23].

In this study, we revised the Berger-HSS [35] to evaluate perceptions of HCV-related stigma among patients. The exploratory factor analysis supported the construct validity of the scale with 85% of items loading on one of four factors and item-clustering matching the Berger subscales for all items that sufficiently loaded on a factor. Furthermore, the Cronbach α results for the overall scale and subscales all exceeded 0.70, supporting internal consistency reliability.

Additionally, our analyses indicated that the common variance of the items is explained most by a single underlying factor (i.e., stigma). The large ratio of the 1^st^/2^nd^ eigenvalues, strong inter-factor correlations, and very high alpha support the unidimensionality of the scale. These findings are consistent with the analyses by Berger et al [35], who found similar inter-factor correlations and the emergence of a single factor solution during scale development [35]. Additionally, we found that the single factor solution explained a comparable proportion of the variance, compared to the four-factor solution (51.8% vs 59.0%). The practical conclusion from these findings is that investigators should use caution in calculating HCV-SS subscale scores as the highly correlated domains measure similar aspects of patients’ experience; the global score of the 33-item one-factor scale is arguably more interpretable.

The unidimensionality of the scale permitted IRT analysis of individual items’ discriminative ability and utility across a wide range of trait levels (item difficulty). The IRT analyses demonstrated that most items had moderate to very high discrimination. Moreover, all but five items showed no measurement invariance between HCV-monoinfected participants and HCV/HIV-coinfected participants, supporting the revision of the Berger HIV-SS for use among patients with HCV infection. IRT-based CAT simulation allowed shortening of the scale while maintaining structural validity and reliability. We found that a few questions accounted for the majority of information provided by the HCV-SS, while several items provided no information across ability levels. The CAT simulation demonstrated low measurement error (SEM<0.3) after only 6 items and acceptable measurement error (SEM<0.4) after only 3 items. The use of polytomous items to measure a narrow construct allowed substantial shortening of the scale while maintaining acceptable test information. These findings support the use of these shorter assessments as practical alternatives to the overall scale, which can reduce survey burden while preserving validity.

This study had several strengths. To our knowledge, this is the first study to validate a scale to specifically measure perceptions of stigma among patients with HCV. By using ACASI software across several sites, including within a syringe-service program, we were able to include participants with low literacy and active drug use, increasing the generalizability of our study. Third, by employing IRT in addition to EFA, we were able to evaluate the psychometric properties of individual items and develop alternative shortened scales.

This study also had several potential limitations. First, while the CAT simulations demonstrate promise to substantially shorten the scale, we did not administer this shortened scale to study participants. To assess its reliability and validity more directly, we plan to administer the shortened scale in a separate sample in future research. Secondly, the sample size used here (N = 265) is relatively small for IRT analyses; however, the large number of items and item fit statistics indicate a stable model. Third, the results here indicate that this scale is measuring a narrow construct, and therefore one could argue that there are aspects of patients’ experiences of HCV stigma that are not being captured by the items. Qualitative research among patients with HCV may inform additional questions to better asses the breadth of patient experiences with HCV stigma. Fourth, since our study is the first to validate a measure of HCV-related stigma, assessment of concurrent validity was not possible due to the lack of availability of other validated HCV stigma instruments. Finally, since participants in our study were recruited from a mainly urban setting, the results might not be generalizable to individuals who reside in non-urban areas. Future analyses should examine the validity of the HCV-SS in those settings.

In conclusion, we found that the revised Berger-HSS has sufficient validity and reliability to measure HCV-related stigma. Validation of this measure has potential utility in evaluating interventions to reduce HCV-related stigma, as well as practical implications for future research on stigma as a barrier to healthcare.

## Supporting information

S1 Appendix Hepatitis C Virus (HCV) Stigma Scale. 33-item Hepatitis C virus Stigma Scale (HCV-SS), administered on audio-computer assisted self-interview software. All items included response options “I don’t know the answer” and “I don’t want to answer.”
(DOC)
