# Supplementary material for: Validation of a modified Berger HIV stigma scale for use among patients with hepatitis C virus (HCV) infection
Source: PLoS One. 2020 Feb 5;15(2):e0228471. doi: 10.1371/journal.pone.0228471 (PMC7001940; doi:10.1371/journal.pone.0228471)
Supplement: S1 Appendix — 33-item Hepatitis C virus Stigma Scale (HCV-SS), administered on audio-computer assisted self-interview software. All items included response options “I don’t know the answer” and “I don’t want to answer.” (DOC) [file pone.0228471.s001.doc]

**S1 Appendix: Hepatitis C Virus (HCV) Stigma Scale**

**Introduction text for this section:**

This section asks about some of the social and emotional aspects of having hepatitis C virus (HCV). For each statement or question, please select the response that best represents your experience with hepatitis C.

Please do your best to answer each question. If you would prefer not to answer a question, please select “Prefer not to answer.” There are no right or wrong answers.

**Stigma Scale Section 1:**

The following 23 questions ask about some of your experiences, feelings, and opinions as to how people with hepatitis C (HCV) feel and how they are treated. Please do your best to answer each question.

1. People's attitudes about hepatitis C make me feel worse about myself

Strongly Disagree Disagree Agree Strongly Agree

2. Telling someone I have hepatitis C is risky

Strongly Disagree Disagree Agree Strongly Agree

3. People with hepatitis C lose their jobs when their employers find out

Strongly Disagree Disagree Agree Strongly Agree

4. I work hard to keep my hepatitis C a secret

Strongly Disagree Disagree Agree Strongly Agree

5. I feel I am not as good a person as others because I have hepatitis C

Strongly Disagree Disagree Agree Strongly Agree

6. People with hepatitis C are treated like outcasts

Strongly Disagree Disagree Agree Strongly Agree

7. Most people believe that a person who has hepatitis C is dirty

Strongly Disagree Disagree Agree Strongly Agree

8. It is easier to avoid new friendships than worry about telling someone that I have hepatitis C

Strongly Disagree Disagree Agree Strongly Agree

9. Since learning I have hepatitis C, I feel set apart and isolated from the rest of the world

Strongly Disagree Disagree Agree Strongly Agree

10. Most people think that a person with hepatitis C is disgusting

Strongly Disagree Disagree Agree Strongly Agree

11. Having hepatitis C makes me feel that I'm a bad person

Strongly Disagree Disagree Agree Strongly Agree

12. Most people with hepatitis C are rejected when others find out

Strongly Disagree Disagree Agree Strongly Agree

13. I am very careful who I tell that I have hepatitis C

Strongly Disagree Disagree Agree Strongly Agree

14. Some people who know I have hepatitis C have grown more distant

Strongly Disagree Disagree Agree Strongly Agree

15. Since learning I have hepatitis C, I worry about people discriminating against me

Strongly Disagree Disagree Agree Strongly Agree

16. Most people are uncomfortable around someone with hepatitis C

Strongly Disagree Disagree Agree Strongly Agree

17. Having hepatitis C in my body is disgusting to me

Strongly Disagree Disagree Agree Strongly Agree

**Stigma Scale Section 2**

Many of the questions in this next section assume that you have told other people that you have hepatitis C, or that others know. This may not be true for you. If the item refers to something that has not actually happened to you, please imagine yourself in that situation. Then select your answer ("strongly disagree," "disagree," "agree," "strongly agree") based on how you think you would feel or how you think others would react to you.

18. I have been hurt by how people reacted to learning I have hepatitis C

Strongly Disagree Disagree Agree Strongly Agree

19. I worry that people who know I have hepatitis C will tell others

Strongly Disagree Disagree Agree Strongly Agree

20. I regret having told some people that I have hepatitis C

Strongly Disagree Disagree Agree Strongly Agree

21. As a rule, telling others that I have hepatitis C has been a mistake

Strongly Disagree Disagree Agree Strongly Agree

22. Some people avoid touching me once they know I have hepatitis C

Strongly Disagree Disagree Agree Strongly Agree

23. People I care about stopped calling after learning I have hepatitis C

Strongly Disagree Disagree Agree Strongly Agree

24. People have told me that getting hepatitis C is what I deserve for how I lived my life

Strongly Disagree Disagree Agree Strongly Agree

25. Some people close to me are afraid others will reject them if it becomes known that I have

hepatitis C

Strongly Disagree Disagree Agree Strongly Agree

26. People don't want me around their children once they know I have hepatitis C

Strongly Disagree Disagree Agree Strongly Agree

27. People have physically backed away from me when they learn I have hepatitis C

Strongly Disagree Disagree Agree Strongly Agree

28. Some people act as though it's my fault I have hepatitis C

Strongly Disagree Disagree Agree Strongly Agree

29. I have stopped socializing with some people because of their reactions to my having hepatitis C

Strongly Disagree Disagree Agree Strongly Agree

30. I have told people close to me to keep the fact that I have hepatitis C a secret

Strongly Disagree Disagree Agree Strongly Agree

31. People who know I have hepatitis C tend to ignore my good points

Strongly Disagree Disagree Agree Strongly Agree

32. People seem afraid of me once they learn I have hepatitis C

Strongly Disagree Disagree Agree Strongly Agree

33. When people learn you have hepatitis C, they look for flaws in your character

Strongly Disagree Disagree Agree Strongly Agree

**Free text question:**

Is there anything else that you would like to share with us about your experience with hepatitis C virus (HCV)? You can also include any comments about the previous questions. *(optional)*
